# Role of Epstein-Barr Virus in Pathogenesis and Racial Distribution of IgA Nephropathy

**DOI:** 10.3389/fimmu.2020.00267

**Published:** 2020-02-28

**Authors:** Katerina Zachova, Petr Kosztyu, Josef Zadrazil, Karel Matousovic, Karel Vondrak, Petr Hubacek, Bruce A. Julian, Zina Moldoveanu, Zdenek Novak, Klara Kostovcikova, Milan Raska, Jiri Mestecky

**Affiliations:** ^1^Department of Immunology, Faculty of Medicine and Dentistry, University Hospital Olomouc, Palacky University Olomouc, Olomouc, Czechia; ^2^Department of Internal Medicine III Nephrology, Rheumatology and Endocrinology, University Hospital Olomouc, Palacky University Olomouc, Olomouc, Czechia; ^3^Department of Medicine, Second Faculty of Medicine, University Hospital Motol, Charles University, Prague, Czechia; ^4^Department of Pediatrics, Second Faculty of Medicine, Charles University, Prague, Czechia; ^5^Department of Medical Microbiology, Second Faculty of Medicine, University Hospital Motol, Charles University, Prague, Czechia; ^6^Department of Medicine, University of Alabama at Birmingham, Birmingham, AL, United States; ^7^Department of Microbiology, University of Alabama at Birmingham, Birmingham, AL, United States; ^8^Department of Surgery, University of Alabama at Birmingham, Birmingham, AL, United States; ^9^Laboratory of Cellular and Molecular Immunology, Institute of Microbiology, Czech Academy of Sciences, Prague, Czechia

**Keywords:** IgA, EBV-Epstein-Barr virus, racial distribution, IgA nephropathy, mucosal immunology

## Abstract

IgA nephropathy (IgAN) is the dominant type of primary glomerulonephritis worldwide. However, IgAN rarely affects African Blacks and is uncommon in African Americans. Polymeric IgA1 with galactose-deficient hinge-region glycans is recognized as auto-antigen by glycan-specific antibodies, leading to formation of circulating immune complexes with nephritogenic consequences. Because human B cells infected *in vitro* with Epstein-Barr virus (EBV) secrete galactose-deficient IgA1, we examined peripheral blood B cells from adult IgAN patients, and relevant controls, for the presence of EBV and their phenotypic markers. We found that IgAN patients had more lymphoblasts/plasmablasts that were surface-positive for IgA, infected with EBV, and displayed increased expression of homing receptors for targeting the upper respiratory tract. Upon polyclonal stimulation, these cells produced more galactose-deficient IgA1 than did cells from healthy controls. Unexpectedly, in healthy African Americans, EBV was detected preferentially in surface IgM- and IgD-positive cells. Importantly, most African Blacks and African Americans acquire EBV within 2 years of birth. At that time, the IgA system is naturally deficient, manifested as low serum IgA levels and few IgA-producing cells. Consequently, EBV infects cells secreting immunoglobulins other than IgA. Our novel data implicate Epstein-Barr virus infected IgA^+^ cells as the source of galactose-deficient IgA1 and basis for expression of relevant homing receptors. Moreover, the temporal sequence of racial-specific differences in Epstein-Barr virus infection as related to the naturally delayed maturation of the IgA system explains the racial disparity in the prevalence of IgAN.

## Introduction

The most common cause of glomerulonephritis in the world, Immunoglobulin A nephropathy (IgAN), is an autoimmune disease in which IgA1-containing circulating immune complexes (CIC) deposit in the glomerular mesangium to induce tissue injury, (1–5), culminating in end-stage renal disease in 20–40% of patients within 20 years of the biopsy diagnosis (3). An extensive search for an exogenous antigen of microbial or food origin failed to identify any uniformly involved in the formation of nephritogenic IgA-containing CIC (6). Subsequent CIC and mesangial deposit analyses revealed that the IgA is exclusively of the IgA1 subclass and is polymeric (pIgA) (4, 5). Furthermore, the *O*-glycans in the hinge region of the heavy chains of the pIgA1 are deficient in galactose (Gd-IgA1) and are recognized as auto-antigen by IgG or IgA antibodies to form nephritogenic CIC (1–3, 5, 7–20). In comparison to IgA2, IgA1 has a hinge region with an additional 9-serine and threonine amino acids that are sites for potential attachment of *O*-glycans (21).

Although IgAN is the most common form of glomerulonephritis in many countries (2, 3, 22–26), its prevalence displays a surprisingly unique geographical distribution. The disease is common in East Asia, Europe, Australia, and North America, but is rare in central Africa and uncommon in African Americans (2, 3, 25–41). Environmental and genetic factors have been considered in these remarkable geographically related differences (24–26).

Studies concerning IgA structure, metabolism, tissue distribution of cells producing IgA1 or IgA2, and Epstein-Barr virus (EBV)-induced differentiation of B cells into IgA-secreting cells (42–47) suggested possible involvement of EBV in the pathogenesis of IgAN. Thus, *in vitro* EBV-transformed peripheral-blood cells from healthy individuals produce almost exclusively pIgA of the IgA1 subclass (42, 45, 46, 48). Importantly, we have demonstrated that EBV-transformed cells from IgAN patients secrete Gd-IgA1 (15). Therefore, we analyzed the Ig isotypic association and phenotypes of *in vivo* EBV-infected B cells from patients with IgAN or other renal diseases, and White and African American healthy controls.

EBV infects most African Black and African American children by age 1–2 years (49–53). At this age, the entire IgA system is naturally deficient, as evidenced by the virtual absence or low levels of serum IgA and paucity of IgA-producing cells in various tissues (54–58). As a marker of IgA system maturation, serum IgA levels reach adult levels in puberty (54, 57). Therefore, the greatly reduced number of IgA^+^ B cells in early childhood diminishes the chance for EBV to infect such cells. In the White population, EBV infection occurs mainly at adolescence and >95% of adults are EBV-infected (51, 52, 59–63). In healthy adult Whites, EBV genomes have been detected in 80–90% of circulating B cells with IgA on their cell surfaces (s; sIgA^+^) (64).

EBV infection has been associated with highly diverse human diseases of infectious (infectious mononucleosis), malignant (nasopharyngeal carcinoma, Burkitt's lymphoma, and Hodgkin's disease), and autoimmune (systemic lupus erythematosus, multiple sclerosis, and inflammatory bowel disease) nature (59–63, 65–70). Previous studies of the role of EBV in IgA production (42, 44, 45, 47, 48) support the participation of EBV in the pathogenesis of IgAN. Consequently, we analyzed EBV-infected B cells from White IgAN patients, Whites with other renal diseases, and White and African American healthy controls for cell-surface (s) Ig isotypes, production of Gd-IgA1 after polyclonal stimulation, and expression of receptors involved in selective homing to various mucosal or systemic lymphoid tissues (71).

## Materials and Methods

### Reagents

All chemicals, unless otherwise specified, were purchased from Sigma (St. Louis, MO). Tissue-culture media and media supplements were purchased from Invitrogen (Carlsbad, CA).

### Study Subjects

Informed consent was obtained from all participants. The ethical committee of the University Hospital in Olomouc and University Hospital in Motol and the UAB Institutional Review Board, protocol #140108002, approved this study. White adult patients with IgAN and non-IgAN kidney disease and White adult healthy controls were recruited at the Nephrology, Rheumatology, and Endocrinology Department and Department of Transfusion Medicine, University Hospital Olomouc and the Department of Medicine of the University Hospital Motol, Czech Republic. The diagnosis of IgAN for 31 patients had been based on staining for IgA as the dominant or co-dominant Ig in the mesangial immune deposits by routine immunofluorescence microscopy of clinically indicated kidney biopsies, in the absence of clinical or laboratory features of nephritis of SLE, IgA vasculitis, or liver disease. The disease-control group was comprised of 20 patients with non-IgAN kidney disease. Baseline clinical data, including gender, age, blood pressure, serum creatinine level, eGFR, urinary albumin/creatinine ratio (ACR), 24-h proteinuria, hematuria, and treatment with angiotensin-converting enzyme (ACE) inhibitor or angiotensin receptor blocker (ARB), were obtained from review of medical records. The healthy-control groups consisted of 59 Whites for the serology studies and 22 Whites for the EBER^+^ studies, and 11 African American adults recruited at the University of Alabama at Birmingham. Biochemical and physical parameters were determined based on routine clinical laboratory analyses in the respective hospitals. The clinical and biochemical characteristics of the study participants are compiled in Table 1.

**Table 1:** Clinical and biochemical characteristics of study subjects.

	**Analysis**	**Number of subjects**	**Sex ratio (M/F)**	**Age (years)**	**Serum IgA concentration (mg/mL)**	**Gd-IgA1 (μg/mL)**	**Gd-IgA1/IgA ratio (μg/mg)**	**Blood pressure (mmHg)**	**eGFR (mL/min per 1.73m^**2**^)**	**Proteinuria (g/day)**	**Urine albumin/creatinine (mg/mmol)**	**Hematuria (Ery./μL)**	**ACEi//ARB (treated/all, %)**
IgAN	EBER^*^	31	18/13	49.0	2.82	8.9	3.2	127/80	61	1.2	43.3	16.0	88.9
Non IgAN^**^	EBER^*^	20	7/13	53.0	1.8	3.1	1.7	130/72	42	1.43	100.0	3.5	75
Healthy White adults	Serology	59	8/51	41.0	1.4	4.2	3.0	ND	ND	ND	ND	ND	0
	EBER^*^	22			1.7	4.7	2.8	ND	ND	ND	ND	ND	0
Healthy African American adults	EBER/Serology	11	0/11	36.5	2.9	5.1	1.8	ND	ND	ND	ND	0	0

*Each cohort is characterized by median values*.

*ACEi, angiotensin converting enzyme inhibitor; ARB, angiotensin receptor blocker; EBER, Epstein-Barr virus encoded RNA; Ery, erythrocyte; Gd-IgA1, galactose-deficient IgA1; IgAN, IgA nephropathy; M/F, male/female; ND, not determined*.

* *EBER represents the cohort of subjects analyzed for B cells*.

** *Idiopathic membranous nephropathy, focal segmental glomerulosclerosis, idiopathic nephrotic syndrome, rheumatoid arthritis with proteinuria, systemic lupus erythematosus nephritis, polymyalgia rheumatica with nephritis, Goodpasture syndrome. Serum Gd-IgA1 concentration was measured using Gd-IgA1 assay kit from Immuno-Biological Laboratories Co., Ltd., Fujioka, Japan*.

### Blood Samples

Peripheral blood mononuclear cells (PBMC), isolated from heparinized blood by centrifugation on Histopaque 1077, were used for all cellular analyses. Isolated PBMC were processed immediately or frozen at −80°C in 20% RPMI 1640, 70% FBS, 10% DMSO for later experiments. Sera were isolated from venous blood drawn into Vacuette serum clot activator tubes.

### Pokeweed Mitogen Stimulation

PWM stimulation was performed by incubating PBMC in complete growth medium composed of RPMI 1640 medium supplemented with 10% FBS and PWM at concentration 5 μg/ml. Cells were grown for 7 days at 37°C in a humidified-CO_2_ atmosphere.

### Cell Staining

Unstimulated or PWM-stimulated PBMCs were stained with fluorochrome-labeled antibodies and oligo DNA probes in successive steps: First, after blocking of cellular surface proteins with PBS (Biosera, Nuaille, France) + 10% FBS, surface-exposed receptors were labeled with combinations of selected fluorochrome-conjugated monoclonal antibodies (mAbs): anti-CD19-PE, anti-CD19-PE-eFluor610, anti-CD19-APC-eFluor780, anti-CD27-PE-Cy7, anti-CD38-PE-Cy5, anti-CD138-PE-Cy7, anti-β1 integrin- PE-Cy7, anti-β7 integrin-FITC, anti-CD62L-PE-Cy5, anti-CCR7-PE-Cy7, anti-CCR9-PE, anti-IgM-FITC, anti-IgD-FITC, Fixable Viability Dye-APC-eFluor780, and Fixable Viability Dye-eFluor450 (e-Bioscience, San Diego, CA), anti-CD21-PE-Cy7, anti-CD27-APC-H7, anti-CCR5-PE-Cy5, anti-IgG-PE-Cy5, anti-CCR10-PE, and anti-α4 integrin-PE-Cy5 (BD Biosciences, San Jose, CA), and anti-IgA-biotin or anti-IgG-biotin (Jackson ImmunoResearch, West Grove, PA) diluted in PBS + 10% FBS. Cells were incubated with various combinations of antibodies (Tables 2A,B) for 20 min at RT in the dark at dilutions optimized in preliminary experiments for each mAb. Biotinylated mAbs were stained using Streptavidin-FITC (Southern Biotech, Birmingham, AL) or Streptavidin—Pacific Orange (ThermoFisher Scientific, Waltham, MA). Conjugated mAbs were selected based on their stability during subsequent hybridization. After staining, the cells were washed and analyzed by flow cytometry directly or fixed before intracellular staining and/or hybridization using 4% paraformaldehyde (EMS, Hatfield, PA) for 10 min at RT, followed by permeabilization with 0.5% Tween 20 (Serva, Heidelberg, Germany) in PBS for 10 min at RT. All gating steps settled were based on the FMO (fluorochromes minus one) controls (Figure S1). The EBER probe specificity was based on Daudi (EBER positive) and Jurkat (EBER negative) cell line.

**Table 2:** **(A)** Usage of fluorophore-labeled antibody used by FACS Canto II flow cytometer; **(B)** Usage of fluorophore-labeled antibodies used by SONY SP6800 flow cytometer.

**Option 1**		**Option 2**		**Option 3**		**Option 4**	
**Fluorophore**	**Antigen**	**Fluorophore**	**Antigen**	**Fluorophore**	**Antigen**	**Fluorophore**	**Antigen**
**(A)**							
FITC	IgA, IgG	FITC	IgA, IgG	FITC	IgA, IgG	FITC	IgA, IgG
PE	CD19	PE-eF610	CD19	Cy3	EBER	PE	CCR10
PE-cy7	CCR9	PE-cy7	CCR7	PE-cy5	CCR5	PE-cy7	CD27
Cy5	EBER	Cy5	EBER	APC-H7	CD19	Cy5	EBER
APC-H7	CD27	APC-H7	CD27			APC-H7	CD27
**(B)**							
Pacific Orange	IgA	Pacific Orange	IgA or IgG	Pacific Orange	IgA	Pacific Orange	IgA
FITC	β7 integrin	FITC	IgM or IgD	PE-cy7	CD21	FITC	IgM or IgD
PE-cy7	β1 integrin	PE-cy7	CD138	PE-cy5	CD62L	PE-cy5	IgG
PE-cy5	α4 integrin	PE-cy5	CD38	Cy3	EBER	Cy3	EBER
Cy3	EBER	Cy3	EBER	PE-eFluor610	CD19	PE-eFluor610	CD19
PE-eFluor610	CD19	PE-eFluor610	CD19	eFluor450	FVD	eFluor450	FVD
		eFluor450	FVD			PE-cy7	CD27

*We have determined that only few fluorophores are stable enough to resist 42°C incubation and high salt concentration, particularly FITC, PE, PE-eFluor610, PE-cy5, PE-cy7, cy3, APC-H7, APC-eFluor780, eFlour450, and Pacific Orange*.

*BD Canto II cytometer was equipped with blue and red laser*.

*SONY SP6800 Spectral cell analyzer was equipped with blue and violet laser*.

Surface-stained, fixed, and permeabilized cells were stained for intracellular immunoglobulins using biotin-labeled anti-IgA or anti-IgG and Streptavidin-FITC or Streptavidin—Pacific Orange, or anti-IgG-PE-Cy5 or anti-IgM-FITC or anti-IgD-FITC in PBS + 0.25% Tween 20 + 5% FBS for 30 min at RT in the dark. Finally, cells were washed with adapting buffer (31.25 mM NaCl, 6.25 mM Na_2_EDTA, 62.5 mM Tris-HCl pH 7.5 and 37.5% formamide) and hybridized with three Cy5- or Cy3-conjugated DNA probes specific for EBER-1 (5′-AAACATGCGGACCACCAGCTGGTAC-3′, 5′-AAGACGGCAGAAAGCAGAGTCTGGG-3′, 5′-AAACCTCTAGGGCAGCGTAGGTCCT-3′ Generi Biotech, Hradec Kralove, Czech Republic) in hybridization solution (10% dextran sulfate, 10 mM NaCl, 30% formamide, 0.1% sodium pyrophosphate, 0.2% polyvinylpyrrolidone, 5 mM Na_2_EDTA, and 50 mM TRIS-HCl, pH7.5). Cells were incubated with probes at concentration 12 pmol/ml for 1 h at 42°C in the dark. At the end, cells were washed with 0.5% Tween 20 in PBS for 10 min and subsequently for 30 min at 42°C, and analyzed by flow cytometry.

### Flow Cytometry Analysis

FACS analyses were performed using Canto II cytometer (BD Biosciences) equipped with blue (488 nm) and red (633 nm) lasers and DIVA software or using SONY Spectral Analyzer (SONY Biotechnologies, San Jose, CA) equipped with blue (488 nm) and violet (405 nm) lasers and SP6800 software. More detailed analyses were performed by FlowJo software (Tree Star Inc., Ashland, OR).

### ELISA Determination of IgA and Gd-IgA1 Levels

ELISA measurement of serum levels of total IgA and Gd-IgA1 was done according to previously described protocols (15, 16). Serum Gd-IgA1 level was measured using a Gd-IgA1 Assay (Immuno-Biological Laboratories Co., Ltd., Fujioka, Japan) according to manufacturer's instructions. Briefly, tested sera were diluted 1:500 in enclosed buffer and added to wells pre-coated with rat anti-Gd-IgA1 IgG. After the incubation for 1 h at RT, plates were washed and incubated with HRP-conjugated mouse anti-human IgA Ab for 30 min at RT. After washing, plates were developed with 3,3′,5,5′-tetramethylbenzidine (TMB; ThermoFisher Scientific) and absorbance was measured at 450 nm by an ELISA reader.

### EBV Serology

Serum levels and Ig isotypes of antibodies specific for EBV antigens, VCA, EA, and EBNA1, were analyzed using commercial ELISA kits (VIDIA s.r.o., Vestec, Czech Republic) according to manufacturer's instructions. In brief, sera were diluted 1:100 in enclosed buffer and applied on ELISA plates pre-coated with respective antigen. Plates were incubated with serum for 30 min at RT, thereafter washed, and incubated with HRP-conjugated secondary mAb. After 1 h, wells were washed, developed with TMB, and absorbance was measured at 450 nm by an ELISA reader.

### Statistics

Normality was examined by QQ Plots and Kolmogorov-Smirnov test. Statistical analysis was performed utilizing student's *t*-test or by Welch's ANOVA in instances where variances were unequal. Mann–Whitney *U*-test was used in instances where non-parametric test was required. When appropriate, paired *t*-test or non-parametric Wilcoxon Signed Rank test was performed. ANOVA model was used for multiple group comparisons with Tukey's *post-hoc* test. Homogeneity of Variances was tested using Levenes' statistic. If non-parametric comparison was required, Kruskal-Wallis test was used instead with Dunn-Bonferroni *post-hoc* test. All statistical analyses were done by GraphPad Prism v.5 software (GraphPad Software, La Jolla, CA) or SPSS v.25 (IBM Corp, Riverside, CA). A *p* ≤ 0.05 was considered to be significant in two-tail tests. Only the statistically significant differences are denoted in the figures.

## Results

### High Frequency of EBV Infection in CD19^+^ and sIgA^+^ B Cells in IgAN

**Figure 1:**
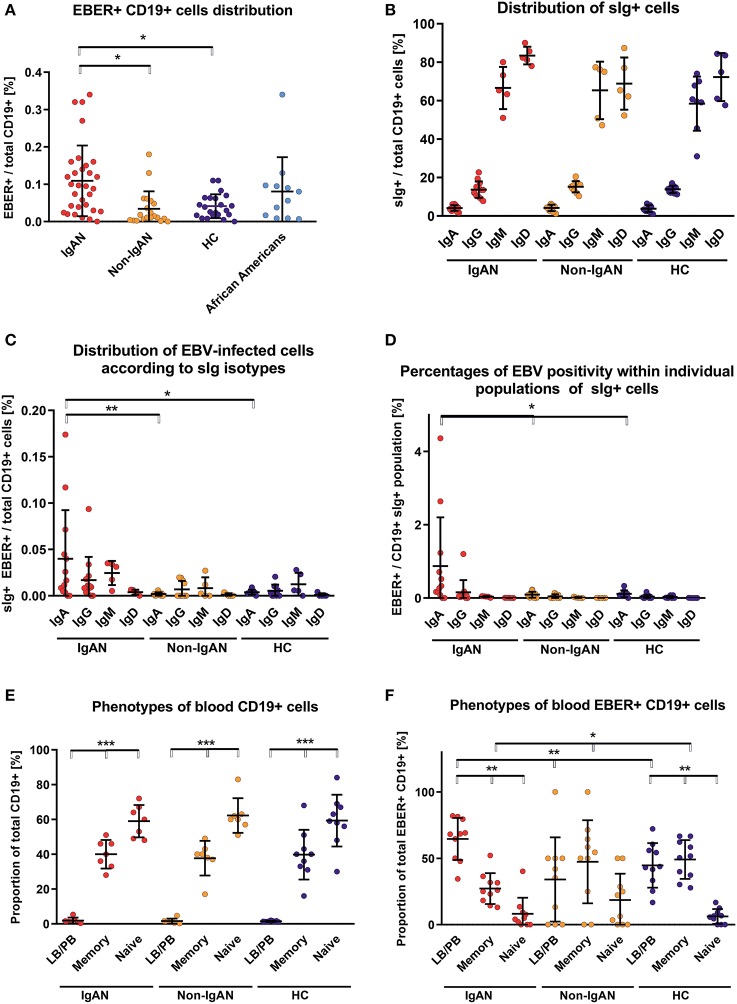
Patients with IgAN exhibit more EBV-infected CD19^+^ cells in peripheral blood than the disease and healthy controls. **(A)** EBER hybridization probe was used to determine the EBV positivity of CD19^+^ cells in PBMC from IgAN patients (IgAN; *n* = 31), non-IgAN patients (non-IgAN; *n* = 20), White healthy controls (HC; *n* = 22), and African American healthy controls (*n* = 11). The highest percentages of EBER^+^ CD19^+^ cells were in the IgAN patients and healthy African Americans. **(B)** Expression of surface (s) immunoglobulins: sIgA, sIgG, sIgM, and sIgD were characterized on CD19^+^ cells from PBMC from IgAN patients (*n* = 12 for IgA and IgG subpopulations and *N* = 5 for IgM and IgD subpopulations), non-IgAN patients (*n* = 10 for IgA and IgG subpopulations and *N* = 5 for IgM and IgD subpopulations), and White healthy controls (*n* = 8 for IgA, IgG, and IgM and *N* = 5 for IgD). **(C)** The proportion of sIgA-, sIgG-, sIgM-, or sIgD-positive EBER^+^ CD19^+^ cells were specified from populations analyzed in panel **(B)** and expressed as percentage of the total pool of CD19^+^ PBMC () or **(D)** as a percentage of isotype-matching CD19^+^ cells. In the panel **(C)** mean percentage values ±SD for IgAN group are IgG = 0.017 ± 0.023, IgM = 0.025±0.01, and for Non-IgAN group are IgG = 0.007 ± 0.008, IgM = 0.008 ± 0.009. In contrast to non-IgAN patients and White healthy controls, in whom EBV was detected preferentially in sIgM and sIgG cells, for IgAN patients the EBER^+^ CD19^+^ cells were predominantly sIgA-positive. **(E)** The distribution of B-cell maturation stages was analyzed in PBMC using surface staining for naive cells (CD19^+^ CD27^−^), memory cells (CD19^+^ CD27^+^), and LB/PB (CD19^dim−^ CD27^++^ CD38^+^). No difference between groups (*n* = 7) in the distribution of individual maturation stages was observed. **(F)** In contrast, analogous populations of EBV-infected (EBER^+^) cells from PBMC of IgAN patients (*n* = 10) exhibited significant changes, namely the dominance of EBER^+^ LB/PB cells and fewer memory B cells when compared to non-IgAN patients (*n* = 10) and White healthy controls (*n* = 10) cohorts. Data are means ± SD. Numerical expression of all presented data (means ± SD) are provided in Supplementary Materials. *P* values were calculated using one-way ANOVA followed by Tukey's *post-hoc* test or using Kruskal-Wallis test with Dunn-Bonferroni *post-hoc* test when needed. *p < 0.05, **p < 0.01, ***p < 0.001.

Using EBV-encoded small RNAs (EBER)-specific probe and phenotypic analysis of CD19^+^ cells, we found that the White EBV-seropositive patients with IgAN displayed a higher frequency of EBV infection (CD19^+^ EBER^+^ cells) compared to non-IgAN patients and White controls (Figure 1A; gating strategy is shown in Figure S1). There were no differences in expression of sIgD, sIgM, sIgG, and sIgA among uninfected CD19^+^ cells between patients with IgAN, patients with non-IgAN kidney diseases, and healthy Whites (Figure 1B). However, the distribution of sIg on EBER^+^ CD19^+^ cells differed between IgAN patients, non-IgAN patients, and White healthy controls. In IgAN patients, these cells were mostly sIgA^+^ rather than sIgM^+^ or sIgG^+^, whereas in the other two groups sIgA^+^ cells were not dominant (Figure 1C). Thus, the percentage of peripheral-blood EBER^+^ CD19^+^ cells related to sIg isotype indicated a preferential association with sIgA^+^ cells for IgAN patients that was not found for non-IgAN patients and White healthy controls (Figure 1D).

To determine additional phenotypic properties of EBER^+^ cells, B cells were classified as naïve, memory, and lymphoblast (LB)/plasmablast (PB) phenotypes according to expression of CD27 and CD38 (naïve B cells are CD19^+^ CD27^−^; memory B cells CD19^+^ CD27^+^; and LB/PB CD19^dim^ CD27^+^ CD38^+^). Although there were few LB/PB cells compared to the numbers of naïve and memory B cells (Figure 1E), LB/PB comprised the majority of the EBER^+^ population from IgAN patients (Figure 1F). These data suggest that more sIgA^+^ cells of LB/PB phenotypes were present in peripheral blood of IgAN patients as compared to non-IgAN patients and White healthy controls.

We performed analogous analysis of EBV-infected cell also for IgAN children and controls (Figure S2). Children with IgAN exhibit moderate increase in EBV-infected CD19^+^ cells in peripheral blood, predominantly of sIgM^+^.

### Paucity of sIgA Expression on EBER^+^ B Cells From Healthy African Americans

**Figure 2:**
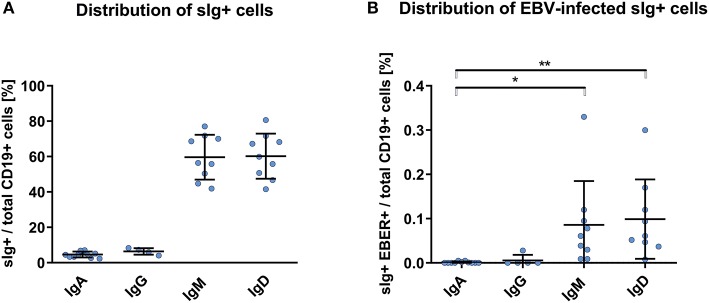
PBMC of African Americans do not exhibit dominance of sIgA^+^ EBV-infected CD19^+^ cells. **(A)** Isotypes of sIg on CD19^+^ PBMC from healthy African Americans (*n* = 9). **(B)** sIg isotype distribution in EBV-infected (EBER^+^) CD19^+^ B cells. This finding contrasts to that for the White healthy controls in whom the fractions of sIgA^+^ and sIgG^+^ cells were more prominent (Figure 1C). Data are means ± SD. Numerical expression of all presented data (means ± SD) are provided in Supplementary Materials. *P* values were calculated using one-way ANOVA followed by Tukey's *post-hoc* test or using Kruskal-Wallis test with Dunn-Bonferroni *post-hoc* test when needed. *p < 0.05, **p < 0.01.

We detected EBER^+^ CD19^+^ cells in blood of the healthy African Americans in frequencies higher, but statistically insignificant, than those in blood from White healthy controls and non-IgAN patients (Figure 1A). Proportions of sIg isotypes of total CD19^+^ cells were similar in IgAN and non-IgAN patients and White and African American healthy controls (Figures 1B, 2A). However, the proportions among the EBV-infected cells markedly differed. In IgAN patients, the cells were mainly sIgA^+^ (Figure 1C), whereas in White healthy controls sIgG^+^ and sIgM^+^ cells dominate with fewer IgA^+^ cells (Figure 1C). In healthy African Americans the EBV-infected cells were dominantly sIgM^+^ and sIgD^+^ with only a negligible contribution from sIgA^+^ cells (Figure 2B). These results are consistent with observations that EBV infection occurs in early childhood more often in African Americans than in Whites.

**Figure 3:**
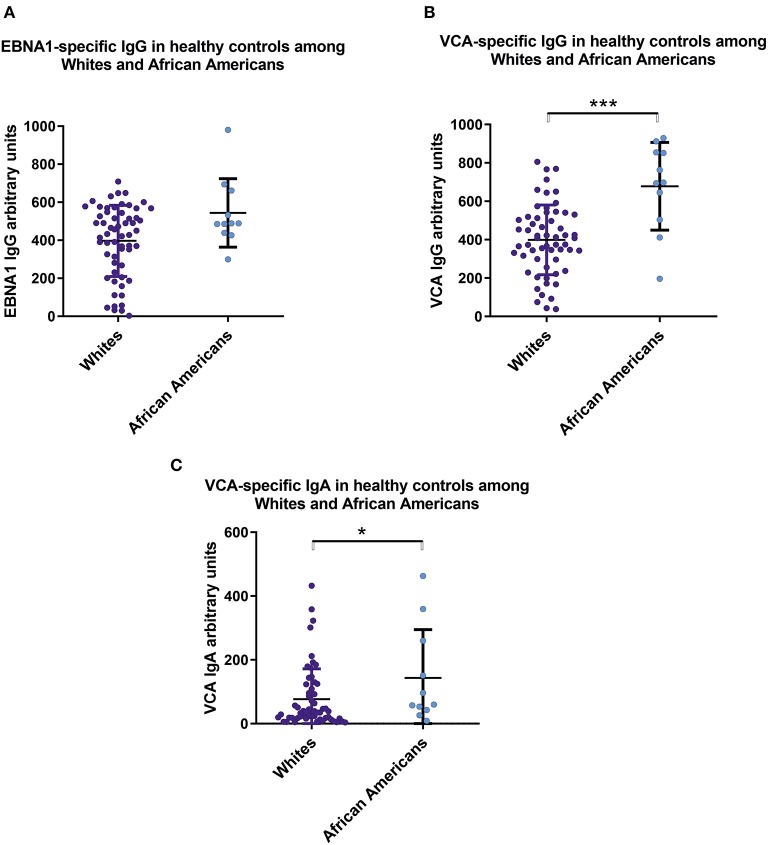
Sera from healthy African Americans exhibit higher levels of IgA and IgG specific for EBV VCA than do sera from White healthy controls. Comparison of serum levels of **(A)** IgG anti-EBNA1, **(B)** IgG anti-VCA, and **(C)** IgA anti-VCA levels in healthy Whites (*n* = 59) and African American (*n* = 11). Particularly, mean values ± SD for IgG anti-ENBA are 396 ± 185 for White healthy controls and 543 ± 171 for African Americans. Numerical expression of all presented data (means ± SD) are provided in Supplementary Materials. In graphs, means and standard deviations are shown. *p < 0.05, ***p < 0.001.

We found significant serological differences between White and African American healthy controls with respect to circulating anti-EBV antibodies. African Americans had higher levels of IgG and IgA specific for viral capsid antigen (Figure 3).

### Expansion of Intracellular IgA^+^ EBER^+^ Cells in IgAN Patients After Polyclonal Stimulation

**Figure 4:**
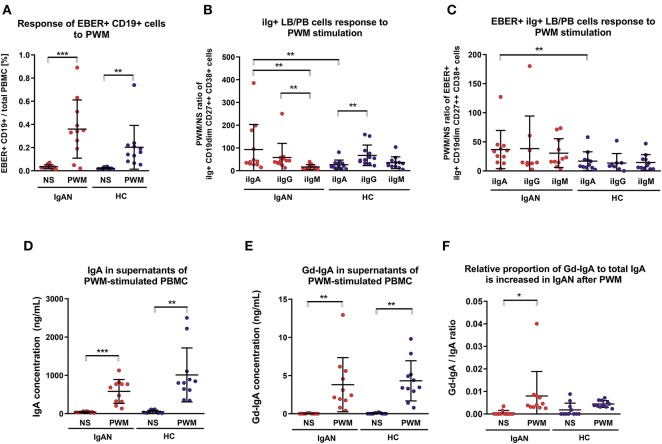
Pokeweed mitogen (PWM) stimulation of PBMC from IgAN patients increases number of intracellular IgA (iIgA^+^) and iIgA^+^ EBER^+^ cells. **(A)** PBMC from IgAN patients (IgAN; *n* = 10) and White healthy controls (HC; *n* = 10) were stimulated with 5 μg/ml PWM for 7 days. Viable cells (Fixable Viability Dye-eFluor negative) were then stained for CD19, CD27, CD38, EBER, and intracellular (i) immunoglobulins: iIgA, iIgG, and iIgM. PWM stimulation significantly increased the proportion of EBER^+^ CD19^dim^ cells compared to cells that were not stimulated (NS), indicating that PWM induced proliferation of EBER^+^ CD19^dim^ cells. **(B)** The proportions of intracellular immunoglobulin isotype iIgA-, iIgG-, and iIgM-positive CD19^dim^ CD27^++^ CD38^+^ cells (lymphoblast/plasmablast; LB/PB) were analyzed before (NS) and after PWM stimulation of PBMC from IgAN patients and White healthy controls. PWM stimulation of PMBC from IgAN patients increased the populations of iIgA^+^, iIgG^+^, and IgM^+^ LB/PB cells in contrast to the increase of iIgM^+^ and iIgG^+^ and less prominent increase of IgA^+^ in PBMC from White healthy controls. **(C)** The proportions of EBER^+^ iIgA^+^, iIgG^+^, or iIgM^+^ in the LB/PB cells were analyzed before and after PWM stimulation of PBMC from IgAN patients and White healthy controls. PWM more effectively stimulated proliferation of iIgA^+^ cells from IgAN patients than cells bearing other Ig isotypes in White healthy controls. **(D)** Measurement of IgA secreted in culture supernatant confirmed a significant increase in total IgA and **(E)** of Gd-IgA1 after PWM stimulation of PBMC from IgAN patients and White healthy controls. The proportion of Gd-IgA1 in the total IgA secreted by PBMC from IgAN patients was moderately, although insignificantly, higher than that secreted by cells from White healthy controls. **(F)** The ratio of Gd-IgA to total IgA was calculated from results in **(D, E)**. Data are means ± SD. Numerical expression of all presented data (means ± SD) are provided in Supplementary Materials. *P* values were calculated using paired *t*-test or non-parametric Wilcoxon Signed Rank test was performed when required. Multiple groups were analyzed using one-way ANOVA followed by Tukey's *post-hoc* test or using Kruskal-Wallis test with Dunn-Bonferroni *post-hoc* test where needed. *p < 0.05, **p < 0.01, ***p < 0.001.

Flares of macroscopic hematuria in IgAN patients are often associated with an acute upper-respiratory-tract infection (2, 3, 37). In the absence of a suitable animal model and ethical restrictions in humans, we examined PBMC after polyclonal and T-cell-dependent stimulation with pokeweed mitogen (PWM) for the number of IgA^+^ EBER^+^ cells. PWM stimulation significantly increased the population of EBV-infected cells from IgAN patients and White healthy controls (Figure 4A). PWM stimulation induced a larger increase in number of intracellular IgA^+^ (iIgA^+^) LB/PB for IgAN patients than for White healthy controls (Figure 4B). For IgAN patients, the response for iIgA^+^ cells was comparable to that for iIgG^+^ cells and exceeded that for iIgM^+^ cells. For White healthy controls, the increase in iIgG^+^ cells exceeded that for iIgA^+^ cells. In contrast, EBER^+^ LB/PB from IgAN patients responded to PWM more than did cells from White healthy controls but a statistical significance was achieved only for iIgA^+^ cells (Figure 4C). For IgAN patients and White healthy controls, PWM-induced secretion of IgA and Gd-IgA1 into the culture supernatants (Figures 4D,E). However, when expressed as ratio of Gd-IgA to total IgA, the response to PWM was significant only for IgAN patients (Figure 4F).

### Altered Expression of Homing Receptors in EBV-Infected B Cells

**Figure 5:**
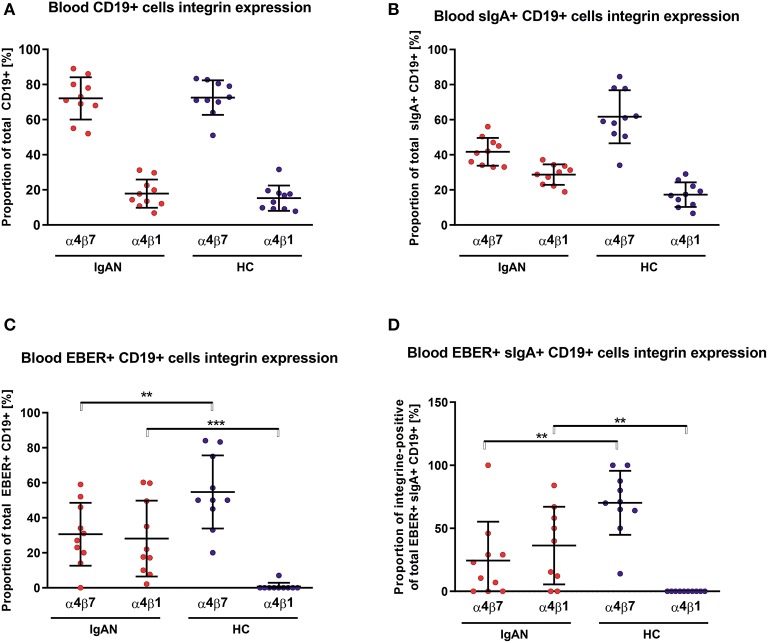
PBMC from IgAN patients contains a substantial proportion of EBER^+^ CD19^+^ α4β1 integrin-positive cells. **(A)** Distribution of integrins α4β7- and α4β1-positive CD19^+^ PBMC was analyzed in IgAN patients (IgAN; *n* = 10) and White healthy controls (HC; *n* = 10). **(B)** Percentage distribution of α4β7 or α4β1 on IgA^+^ CD19^+^ PBMC. Values in percentage are α4β7 = 41.7 ± 7.5 and α4β1 = 28.7 ± 5.5 for IgAN group and α4β7 = 61.7 ± 14.3 and α4β1 =17.3 ± 6.6 for HC **(C)** Percentage distribution of α4β7 or α4β1 on EBV-infected (EBER^+^) CD19^+^ PBMC. **(D)** Percentage distribution of α4β7 or α4β1 on EBV-infected (EBER^+^) CD19^+^ PBMC expressing sIgA. Proportion of α4β7- and α4β1-positive CD19^+^ cells is comparable in IgAN patients and White healthy controls, with significant dominance of α4β7-single-positive CD19^+^ cells, see section A. In contrast to sIgA^+^ CD19^+^ cells from White healthy controls, cells from IgAN patients did not exhibit such an α4β7 dominance, see section B. Moreover, EBV-infected CD19^+^ and, particularly, EBV-infected sIgA^+^ CD19^+^ cells from IgAN patients displayed a reduced proportion of α4β7-positive cells and a larger α4β1-positive subpopulation. In White healthy controls the α4β1-single-positive sIgA^+^ CD19^+^ cells were absent whereas the population of α4β7^+^-single-positive cells was increased, indicating different migration behavior between EBV-infected cells in IgAN patients and White healthy controls. Data are means ± SD. Numerical expression of all presented data (means ± SD) are provided in Supplementary Materials. *P* values were calculated using student's *t*-test or by Welch's ANOVA in instances where variances were unequal. Mann-Whitney *U*-test was used in instances where non-parametric test was required. *p < 0.05, **p < 0.01, ***p < 0.001.

**Figure 6:**
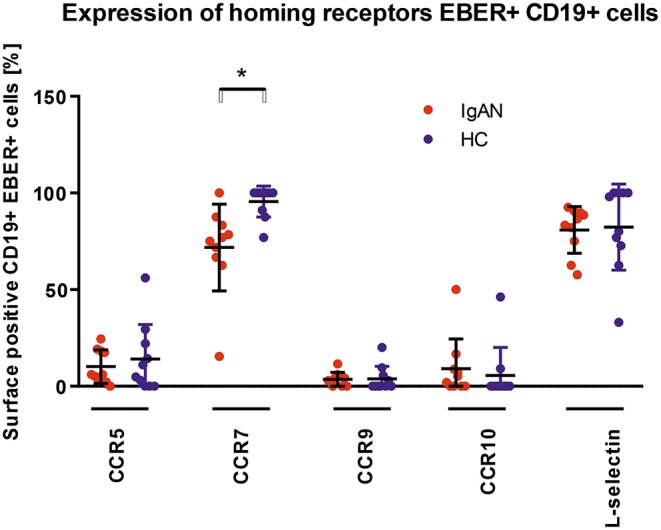
Analysis of changes in expression of trafficking molecules in EBV-infected CD19^+^ PBMC from IgAN and HC. Surface expression of selected trafficking molecules was analyzed in PBMC of IgAN patients (*n* = 10) and HC (*n* = 10) with regard to CD19^+^ EBER^+^ cells. Significant changes were detected for CCR7. Data are means ± SD. Numerical expression of all presented data (means ± SD) are provided in Supplementary Materials. *P* values were calculated using student's *t*-test or by Welch's ANOVA in instances where variances were unequal. Mann–Whitney *U*-test was used in instances where non-parametric test was required. *p < 0.05.

We examined the expression of integrins (L-selectin, α4β1, and α4β7) and relevant chemokine receptors on EBV-negative and -infected B cells and their sIgA^+^ subpopulations from peripheral blood of IgAN patients and White healthy controls. For EBV-negative cells, the numbers of B cells and the ratios of sIgA^+^ subpopulation expressing α4β1^+^ and α4β7^+^ were similar in IgAN patients and White healthy controls (Figures 5A,B). However, analogous comparative studies revealed highly significant differences between these two groups for the expression of homing receptors on EBV-infected cells. Cells from IgAN patients exhibited equal expression of gut-homing receptor α4β7 and respiratory-tract homing receptor α4β1 (Figure 5C) whereas cells from healthy White controls express only gut-homing α4β7 but no α4β1 (Figure 5C). Analogous differences in the expression of homing receptors between IgAN patients and White healthy controls were detected in the sIgA^+^ EBV-infected subpopulation (Figure 5D). In addition to α4β1, these cells displayed enhanced expression of CCR7 (Figure 6) that is involved in homing to systemic and mucosal sites. There was no significant difference in expression of the homing receptors CCR5, CCR9, CCR10, and L-selectin. Therefore, phenotypic analysis of CD19^+^ sIgA^+^ EBER^+^ B cells from patients with IgAN demonstrated a significantly altered expression of homing receptors, with preferential targeting to the upper respiratory tract and tonsils.

## Discussion

Our studies support the involvement of EBV in the pathogenesis of IgAN. We found that B cells and their sIgA^+^ subpopulation in peripheral blood of IgAN patients displayed a significantly higher frequency of EBV infection than did cells of the same phenotype from non-IgAN patients and White healthy controls. Upon polyclonal stimulation *in vitro*, the EBV-infected sIgA^+^ cells secreted Gd-IgA1 that is bound by glycan-specific antibodies to form immune complexes that induce the glomerular injury of IgAN (1–3, 5, 7–20). Although about 40% of first-degree relatives of IgAN patients and some healthy individuals have increased serum Gd-IgA1 levels, most do not have any clinical manifestation of renal disease (1–3, 72–74). It is well established that the quantities and relative stoichiometric proportions of antigen (Gd-IgA1) to antibody (glycan-specific antibody) play the decisive role in formation of nephritogenic immune complexes (1–3).

The EBV-infected sIgA^+^ cells displayed increased expression of homing receptors for targeting the upper respiratory tract. Migration and tissue distribution of lymphocytes and other cell populations depend on expression of integrin and chemokine receptors on cell surfaces and their specific interactions with corresponding ligands on endothelial cells of post-capillary venules (56, 71, 75–79). For sIgA^+^ B cells and LB/PB in peripheral blood and IgA-producing plasma cells in mucosal or systemic lymphoid tissues, precursors of such cells originate in mucosal inductive sites to subsequently populate anatomically remote effector sites (56, 71, 75–79). Expression of homing receptors is profoundly influenced and altered by viruses, including EBV (80–84). The significantly altered expression of homing receptors of the EBV-infected sIgA^+^ cells provides a plausible basis for the common occurrence of synpharyngitic macroscopic hematuria in patients with IgAN.

Our study included healthy African Americans and White healthy controls who displayed key differences in phenotypic characteristics of B cells and serological findings. EBV had infected and remained preferentially associated with sIgM^+^ and sIgD^+^ rather than sIgA^+^ cells. This difference may account for the low frequency of IgAN in African Blacks and African Americans. EBV may infect B cells from the earliest stages of their development and *in vitro* may induce their differentiation into plasma cells which express only intracellular J chain but do not secrete Ig due to the lack of Ig gene rearrangement (85, 86). African Blacks and African Americans are infected with EBV early in childhood, after the protective effect of maternal IgG anti-EBV antibodies acquired passively through placental transport has waned (49, 50, 87–89). Naturally delayed maturation of the IgA system in early childhood manifests as absent or low levels of IgA in sera and secretions and low numbers of IgA-producing cells in tissues (54–58). In this setting, the possibility that EBV will infect such cells is greatly diminished. Instead, EBV infects B cells committed to produce Ig of other isotypes. Due to the ensuing induction of humoral and cellular immune responses in infected children (68, 70, 90–94), EBV is confined to the initially infected B cell population and will not later enter cells that secrete IgA. The mechanisms involved in the asymptomatic EBV infection of children have not been sufficiently explored. Levels of circulating CD8^+^ T cells with robust cytotoxic T cell activity are high in very young children, and then substantially diminish with age (90, 91, 93, 94). Detailed comparative epidemiological studies in Africa and the US (including African Americans and Whites) revealed that these markedly divergent temporal and racial differences in EBV infections are highly correlated with the socio-economic status (hygiene, family size, income, and education), stress, nutrition, co-infection, breastfeeding, magnitude of cellular and humoral immune responses, and genetic background (51–53, 63, 68, 87–89, 92, 95–97). Thus, early asymptomatic EBV infection with ensuing immune responses may be responsible for the well-documented low incidence of IgAN as well as infectious mononucleosis in African Blacks and African Americans (2, 3, 25–46, 48–71, 90, 96).

Another aspect that should be considered with respect to an association of EBV with IgAN concerns the familial incidence of EBV infection (50–52, 59–61, 96). In addition to the probably dominant role of genetic associations with IgAN (2, 3, 26, 97), EBV infection of relatives in close contact may contribute to the clustering of IgAN in some families.

Our results are based on the analyses of B cells from peripheral blood. The virus enters through the oro-pharyngeal mucosa and infects epithelial cells and B cells in Waldeyer's ring, collections of small lymphoid tissues dispersed in the oropharyngeal mucosa, and draining lymph nodes (51, 59–61, 63, 70, 79). Furthermore, Ig-producing cells in the nasopharyngeal and upper respiratory tract mucosae, palatine tonsils, and adenoids contain, in comparison to other mucosal tissues, almost exclusively IgA1-producing cells (46, 56, 79, 81). EBV-infected cells are detectable in B cells in Waldeyer's ring, draining lymph nodes, and peripheral blood, whereas other lymphoid tissues (bone marrow, spleen, and intestinal mucosa) do not contain EBV-infected cells (59–61, 63, 70, 92, 98–100). Because tonsillectomy is infrequently performed for IgAN patients in most countries, the use of B cells from peripheral blood is justifiable on the basis of their highly relevant phenotypic characteristics.

The participation of tonsillar B cells, LB, PB, and plasma cells in the pathogenesis of IgAN remains controversial (101, 102). Although relevant tonsillar abnormalities, including the increased production of polymeric Gd-IgA1, have been reported (103–109) it remains unclear whether the palatine tonsils are a major source of Gd-pIgA1; other compartments of Waldeyer's ring and draining lymph nodes may be involved. Because most of the EBER^+^ sIgA^+^ cells in our studies displayed LB and PB phenotypes, they may also contribute to the circulatory pool of Gd-IgA1.

Increased serum IgA1 levels are common in patients with IgAN (2, 3). Several cytokines participate in isotype switching from sIgM^+^ to sIgA^+^ in B cells and further differentiation to IgA-producing cells (110, 111). TGFβ appears to play the essential role in switching B cells from sIgM^+^ to sIgA^+^, and IL-10 is critical for terminal differentiation into IgA-secreting plasma cells (110–114). Increased serum levels of IL-10 are present in IgAN patients (114–116). As an IL-10 analog is within the EBV genome (117–120), it is plausible that virus-encoded IL-10 (vIL-10) and other EBV-induced immunoregulatory cytokines (121, 122), structurally and functionally homologous to their human counterparts, enhance terminal cellular differentiation and increase IgA production in IgAN patients.

EBV infects nearly all humans worldwide, but most individuals remain without apparent clinical manifestations. In this respect, EBV infection is, in its outcome, reminiscent of infections with other herpes viruses and human papilloma virus (123). In most infected individuals, these viruses establish a lifelong latent residence without associated clinical manifestations. However, in a genetically or immunologically susceptible population, EBV infection may display a highly variable outcome resulting in a broad spectrum of seemingly unrelated diseases (51, 59–63, 65–70, 90–92). We suggest that this spectrum now includes IgAN.

Our study has limitations. First, due to unavailability of relevant samples of blood from African American and African Black children, we could not assess the precise timing of EBV infection. Second, the results cannot be experimentally addressed, due to the unavailability of a relevant animal model. Although hominoid primates, chimpanzee and gorilla, possess IgA1 and IgA2 subclasses highly homologous in their protein and glycan structures, including the hinge region, to their human counterparts (21, 23), they are refractory to EBV infection. The Old World primates are infectable by EBV but produce IgA homologous to human IgA2 that lacks the hinge region with *O*-linked glycans of critical importance in the pathogenesis of IgAN (2, 3).

**Figure 7:**
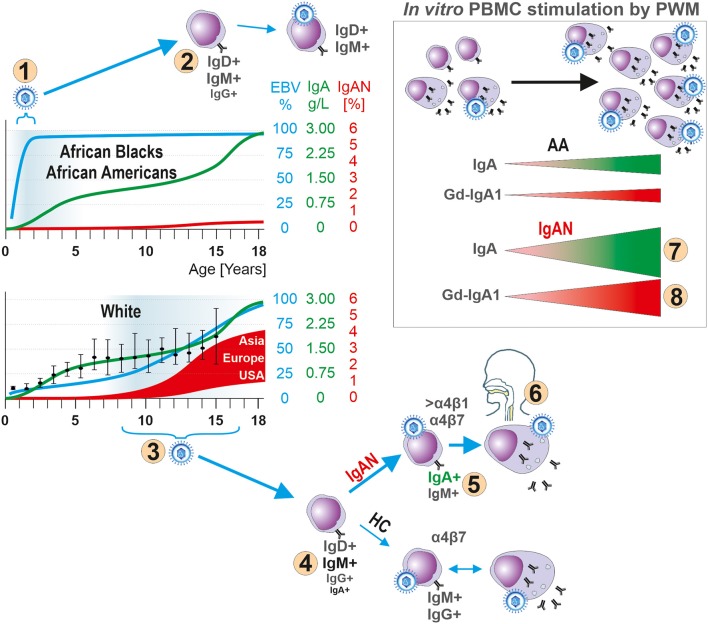
Hypothesis for the role of EBV in the pathogenesis of IgAN. In children, development of the IgA system displays natural, physiologically delayed, maturation as manifested by low levels of IgA in body fluids and few IgA-producing cells in lymphoid tissues. The early EBV infection of African Blacks and African Americans (number “1”) occurs at the time of immaturity of the IgA system, thereby reducing the chance for EBV to infect sIgA^+^ B cells due to their paucity. Instead, sIgM^+^ and sIgD^+^ B cells are preferentially infected (number “2”). The well-described humoral and cellular immune responses to a primary EBV exposure prevent spreading of EBV to other yet uninfected B cells, including sIgA^+^ cells, or infection of sIgA^+^ cells during a subsequent exposure to the virus. In contrast, Whites are infected with EBV at onset or during pubescence (number “3”), when sIgA^+^ cells are present, leading to entry of the virus into B cells, including sIgA^+^ cells (number “4”) which leads to increased synthesis of Gd-IgA1 that can cause IgAN (number “5”). In IgAN patients most EBV^+^ sIgA^+^ cells express Waldeyer's ring- and upper respiratory tract-homing receptor α4β1 (number “6”) thereby targeting the cells to sites where an infection is often associated with an episode of macroscopic hematuria in patients with IgAN. Indeed, *in vitro* PWM stimulation of EBER^+^ CD19^+^ cells from IgAN patients induces proliferation of IgA-producing cells (number “7”) and especially Gd-IgA1-producing cells (number “8”). If a clinical infection were to induce similar effect *in vivo*, the newly formed Gd-IgA1 may be the target for circulating autoantibodies to form immune complexes that deposit in the glomerular mesangium to induce the renal injury of IgAN. HC abbreviates for White healthy controls.

We propose that EBV is intimately involved in the characteristic racial prevalence of IgAN based on an age-dependent initiation of infection. This association may be related to the naturally delayed maturation of the IgA system (Figure 7). This novel concept is supported by the low incidence of IgAN and infectious mononucleosis in geographical locations with convincingly documented differences of EBV infections in very early childhood. Furthermore, the reported similarities in clinical and laboratory findings in patients with IgAN and IgA vasculitis with nephritis (Henoch-Schoenlein purpura nephritis) (3) suggest the possible involvement of EBV also in the latter disease. Our findings suggest that the ongoing efforts to develop vaccines (124–126) against infectious mononucleosis and EBV-associated malignancies may also reduce the incidence of IgAN.

## Data Availability Statement

The datasets generated for this study are available on request to the corresponding authors.

## Ethics Statement

Written informed consent was obtained from all participants. The ethical committee of the University Hospital in Olomouc and University Hospital in Motol and the UAB Institutional Review Board, protocol #140108002, approved this study.

## Author Contributions

JM and MR designed the research and wrote the paper. KZ, PK, ZM, and KK performed research. JZ, KM, KV, ZN, ZM, PH, and BJ performed research and analyzed data.

### Conflict of Interest

BJ is a co-founder and holds equity in Reliant Glycosciences, LLC and has received financial support from IGA Nephropathy Foundation of America, clinical-study grants from Calliditas Pharmaceuticals and Retrophin Inc., a research grant from Alexion Pharmaceuticals, and an honorarium from Visterra Inc. The remaining authors declare that the research was conducted in the absence of any commercial or financial relationships that could be construed as a potential conflict of interest.

## Abbreviations

ACE: Angiotensin-converting enzyme
ARB: Angiotensin receptor blocker
CIC: Circulating Immune Complexes
EBER: EBV-encoded small RNA
EBV: Epstein-Barr virus
Gd-IgA1: Galactose-deficient IgA1
IgA: Immunoglobulin A
IgAN: IgA nephropathy
LB: Lymphoblast
PB: Plasmablasts
PBMC: Peripheral blood mononuclear cells
pIgA: Polymeric IgA
PWM: Pokeweed mitogen
sIgA^+^: Surface IgA-positive B cell
vIL-10: virus-encoded IL-10.
