# Supplementary material for: Role of Epstein-Barr Virus in Pathogenesis and Racial Distribution of IgA Nephropathy
Source: Front Immunol. 2020 Feb 28;11:267. doi: 10.3389/fimmu.2020.00267 (PMC7058636; doi:10.3389/fimmu.2020.00267)
Supplement: Supplementary file 1 [file Data_Sheet_1.pdf]

Figure S1. Gating strategy of EBER$^+$ sIgA$^+$ B lymphocytes and FMO control.

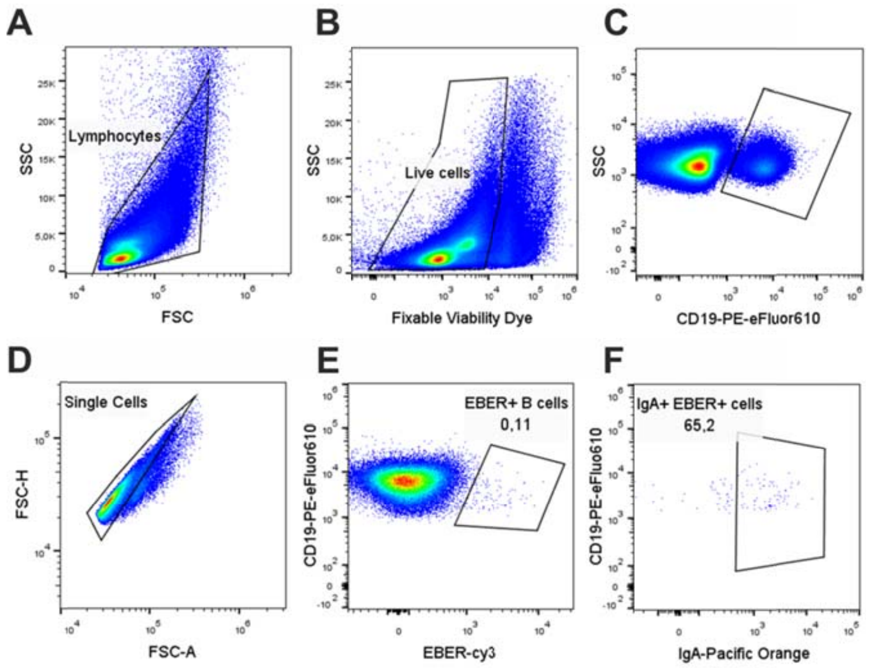

G

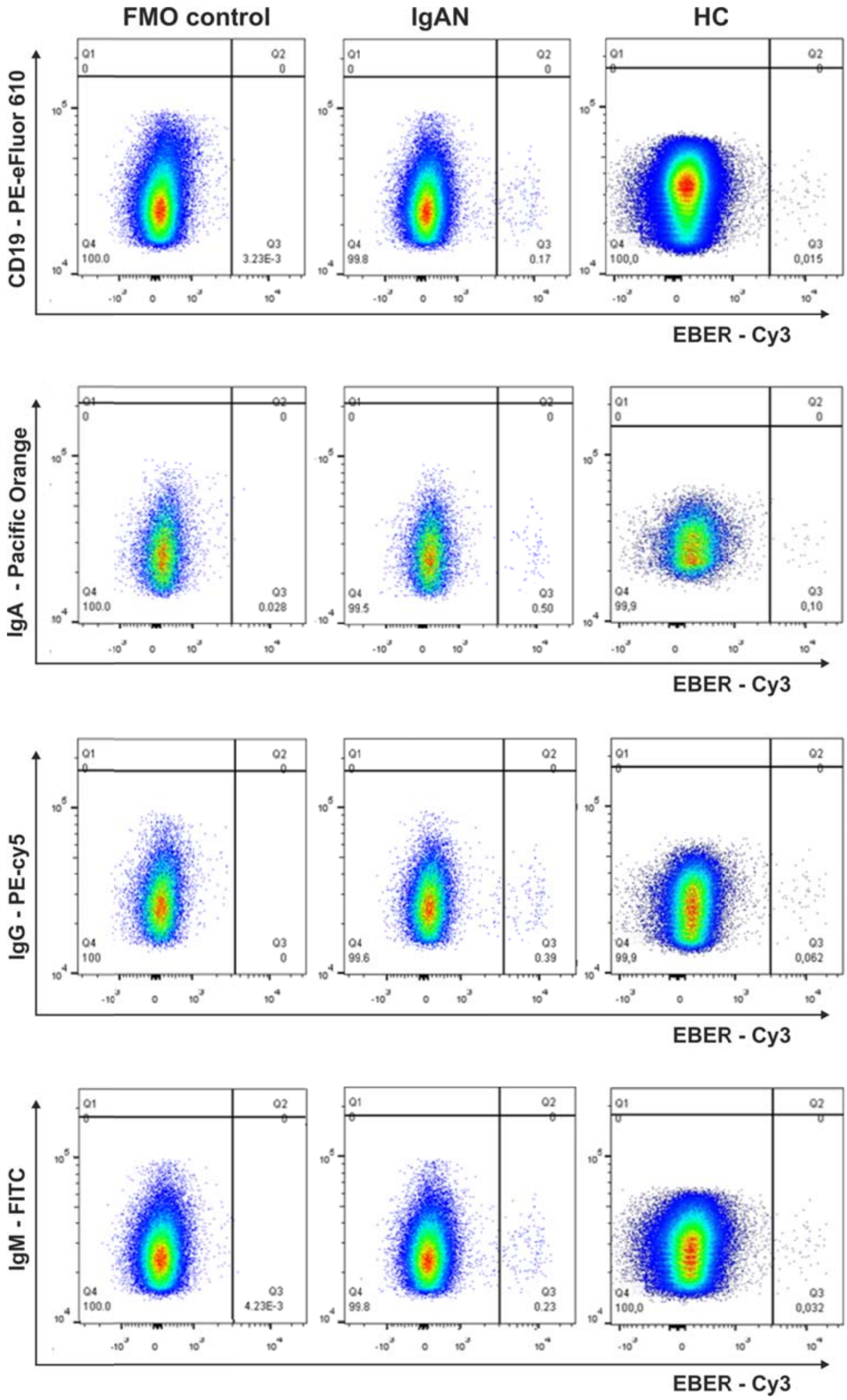

FMO control
IgAN
HC
CD19 - PE-eFluor 610
IgA - Pacific Orange
IgG - PE-cy5
IgM - FITC
EBER - Cy3
Q1
Q2
Q3
Q4
100.0
3.23E-3
99.8
0.17
100,0
0,015
100.0
0.028
99.5
0.50
99,9
0,10
100
0
99.6
0.39
99,9
0,062
100.0
4.23E-3
99.8
0.23
100,0
0,032

(**A**) Initial gate was set on lymphocytes in the forward scatter (FSC) versus side scatter (SSC) dot plot. (**B**) Live cells were identified using Fixable Viability Dye. (**C**) $CD19^+$ B cells were selected. (**D**) Cell doublets were excluded using FSC-A vs FSC-H characteristics. (**E**) $EBER^+$ B lymphocytes or (**F**) $EBER^+$ $sIgA^+$ B lymphocytes were identified. Numbers indicate percentage of paternal population. In total of $2x10^6$ events measured, 115 $EBER^+$ B lymphocytes were identified according to suggested strategy for rare population identification. All gates were set based on FMO (Fluorochrome minus one) controls. (**G**) Gating strategy based on FMO controls. Gates of EBER-positive $CD19^+$ cells, $sIgA^+$ $CD19^+$ B cells, $sIgG^+$ $CD19^+$ cells and $sIgM^+$ $CD19^+$ cells were settled by fluorochrome minus one (FMO) controls.

Figure S2. Children with IgA nephropathy exhibit moderate increase in EBV-infected CD19^{+} cells in peripheral blood predominantly of sIgM.

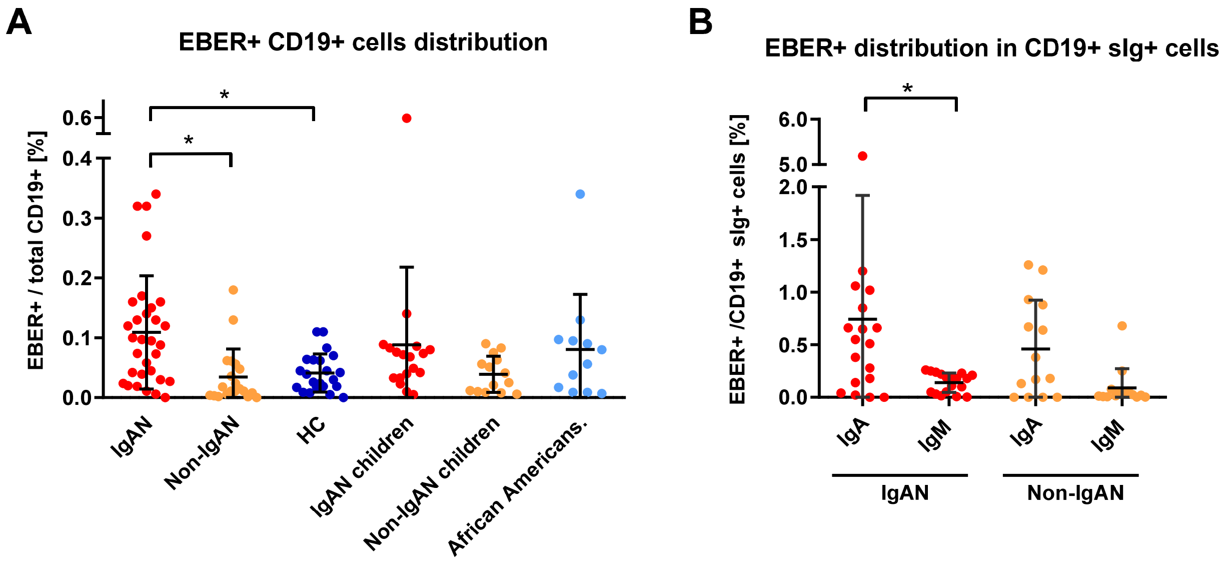

(**A**) EBER hybridization probe was used to determine the EBV positivity of CD19^{+} in PBMC from adult IgAN patients (n=31), adult non-IgAN patients (n=20), White healthy controls (HC) (n=22), pediatric IgAN patients (n=17), pediatric non-IgAN kidney-disease patients (n=14), and healthy African American adults (n=11). Data are means ± SD. P values were calculated using one-way ANOVA followed by Tukey's post-hoc test. * p value <0.05. (**B**) Percentage of EBV-infected sIgA^{+} and sIgM^{+} CD19^{+} PBMC from pediatric patients with IgAN or non-IgAN kidney disease indicates significant dominance of sIgA^{+} cells in IgAN children. The number of EBV-infected cells that were sIgA^{+} exceeded that for sIgM^{+} cells. Data are means ± SD. P values were calculated using one-way ANOVA followed by Tukey's post-hoc test or using Kruskal-Wallis test with Dunn-Bonferroni post-hoc test when needed. * p value <0.05.

Supplementary Table 1. Numerical expression of measured values (mean±SD) shown in Fig.1 through Fig.6

Figure 1. Patients with IgAN exhibit more EBV-infected CD19+ cells in peripheral blood than the disease and healthy controls.

**Fig. 1A**	**EBER+ CD19+ cells distribution**			
EBER+ / total CD19+	IgAN	Non-IgAN	HC	African Americans
[%]	0.109±0.093	0.034±0.045	0.041±0.032	0.081±0.088

**Fig. 1B**	**Distribution of sIg+ cells**											
	IgAN				Non-IgAN				HC			
sIg+ / total CD19+ cells	IgA	IgG	IgM	IgD	IgA	IgG	IgM	IgD	IgA	IgG	IgM	IgD
[%]	4.2±1.4	13.7±4.1	66.6±9.8	83.5±4.1	4.2±1.6	15.2±2.8	65.3±13.4	68.9±12.2	3.8±1.7	13.9±1.8	58.5±13.2	72.3±11.2

**Fig. 1C**	**Distribution of EBV-infected cells according to sIg isotypes**											
	IgAN				Non-IgAN				HC			
sIg+ EBER+ / total CD19+ cells	IgA	IgG	IgM	IgD	IgA	IgG	IgM	IgD	IgA	IgG	IgM	IgD
[0.01%]	4±5	2±2	2±1	0.4±0.2	0.2±0.2	0.7±0.8	0.8±0.99	0.1±0.1	0.4±0.2	0.5±0.6	1.2±1	0.1±0.1

**Fig. 1D**	**Percentages of EBV positivity within individual populations of sIg+ cells**											
	IgAN				Non-IgAN				HC			
EBER+ / CD19+ sIg+ population	IgA	IgG	IgM	IgD	IgA	IgG	IgM	IgD	IgA	IgG	IgM	IgD
[0.1%]	8.7±13	1.6±3.2	0.4±0.2	0.05±0.03	0.9±0.8	0.4±0.5	0.1±0.1	0.02±0.02	1.1±0.9	0.4±0.5	0.3±0.3	0.01±0.02

**Fig. 1E**	**Phenotypes of blood CD19+ cells**								
	IgAN			Non-IgAN			HC		
Proportion of total CD19+	LB/PB	Memory	Naïve	LB/PB	Memory	Naïve	LB/PB	Memory	Naïve
[%]	1.91±1.55	40.0±7.6	59.0±8.6	1.61±1.36	37.71±9.2	62.29±9.2	1.43±0.31	39.78±13.48	59.33±14.05

**Fig. 1F**	**Phenotypes of blood EBER+ CD19+ cells**								
	IgAN			Non-IgAN			HC		
Proportion of total EBER+ CD19+	LB/PB	Memory	Naïve	LB/PB	Memory	Naïve	LB/PB	Memory	Naïve
[%]	64.6±15	27.2±11	8.2±11.5	34.1±30.1	47.4±29.7	18.6±18.8	44.7±15.9	49.1±13.9	6.2±5.3

Figure 2. PBMC of African Americans do not exhibit dominance of sIgA+ EBV-infected CD19+ cells.

**Fig. 2A**	**Distribution of sIg+ cells**			
	IgA	IgG	IgM	IgD
sIg+ / total CD19+ cells [%]	4.62±1.59	6.36±1.58	59.62±11.95	60.24±12.02

**Fig. 2B**	**Distribution of EBV-infected sIg+ cells**			
	IgA	IgG	IgM	IgD
sIg+ EBER+ / total CD19+ cells [%]	0.001±0.002	0.006±0.012	0.086±0.093	0.099±0.085

Figure 3. Sera from healthy African Americans exhibit higher levels of IgA and IgG specific for EBV VCA than do sera from White healthy controls.

**Fig. 3A**	**EBNA1-specific IgG in healthy controls among Whites and African Americans**	
	Whites	African Americans
EBNA1 IgG arbitrary units	396.69±185.56	543.66±171.68

**Fig. 3B**	**VCA-specific IgG in healthy controls among Whites and African Americans**	
	Whites	African Americans
VCA IgG arbitrary units	398.64±180.4	678.03±217.66

**Fig. 3C**	**VCA-specific IgA in healthy controls among Whites and African Americans**	
	Whites	African Americans
VCA IgA arbitrary units	76.74±94.08	143.27±144.27

Figure 4. Pokeweed mitogen (PWM) stimulation of PBMC from IgAN patients increases number of intracellular IgA (iIgA+) and iIgA+ EBER+ cells.

**Fig. 4A**	**Response of EBER+ CD19+ cells to PWM**			
	IgAN		HC	
	NS	PWM	NS	PWM
EBER+ CD19+ / total PBMC [%]	0.03±0.01	0.36±0.24	0.02±0.01	0.20±0.18

**Fig. 4B**	**iIg+ LB/PB cells response to PWM stimulation**					
	IgAN			HC		
	IgA	IgG	IgM	IgA	IgG	IgM
PWM/NS ratio of iIg+ CD19dim CD27++ CD38+ cells	93.18±105.4	58.03±59.58	16.57±9.93	26.49±19.37	67.50±43.30	35.12±24.86

**Fig. 4C**	**EBER+ iIg+ LB/PB cells response to PWM stimulation**					
	IgAN			HC		
	IgA	IgG	IgM	IgA	IgG	IgM
PWM/NS ratio of EBER+ iIg+ CD19dim CD27++ CD38+ cells	36.75±31.24	38.43±52.75	30.60±23.59	16.83±15.41	13.61±15.23	14.58±12.86

**Fig. 4D**	**IgA in superntants of PWM-stimulated PBMC**			
	IgAN		HC	
	NS	PWM	NS	PWM
IgA concentration (ng/mL)	39.40±15.68	580.64±296.58	49.36±37.56	1011.18±673.30

**Fig. 4E**	**Gd-IgA in supernatants of PWM-stimulated PBMC**			
	IgAN		HC	
	NS	PWM	NS	PWM
Gd-IgA concentration (ng/mL)	0.02±0.04	3.81±3.39	0.05±0.07	4.31±2.51

**Fig. 4F**	**Relative proportion of Gd-IgA to total IgA is increased in IgAN after PWM**			
	IgAN		HC	
	NS	PWM	NS	PWM
Gd-IgA / IgA ratio (0.01)	0.05±0.1	0.8±1	0.18±0.29	0.45±0.14

Figure 5. PBMC from IgAN patients contains a substantial proportion of EBER+ CD19+ α4β1 integrin-positive cells.

**Fig. 5A**	**Blood CD19+ cells integrin expression**			
	IgAN		HC	
Proportion of total CD19+	α4β7	α4β1	α4β7	α4β1
[%]	72.1±11.4	17.9±7.6	72.5±9.4	15.3±6.8

**Fig. 5B**	**Blood sIgA+ CD19+ cells integrin expression**			
	IgAN		HC	
Proportion of total sIgA+ CD19+	α4β7	α4β1	α4β7	α4β1
[%]	41.7±7.5	28.7±5.5	61.7±14.3	17.01±6.6

**Fig. 5C**	**Blood EBER+ CD19+ cells integrin expression**			
	IgAN		HC	
Proportion of total EBER+ CD19+	α4β7	α4β1	α4β7	α4β1
[%]	30.6±17.1	28.1±20.6	54.7±19.8	0.7±2.1

**Fig. 5D**	**Blood EBER+ sIgA+ CD19+ cells integrin expression**			
	IgAN		HC	
Proportion of integrine-positive of total EBER+ sIgA+ CD19+	α4β7	α4β1	α4β7	α4β1
[%]	24.5±29.2	36.3±29.0	70.2±24.1	0±0

Figure 6. Analysis of changes in expression of trafficking molecules in EBV-infected CD19+ PBMC from IgAN and HC.

	**Expression of homing receptors EBER+ CD19+ cells**									
	CCR5		CCR7		CCR9		CCR10		L-selectin	
Surface positive CD19+ EBER+ cells [%]	IgAN	HC	IgAN	HC	IgAN	HC	IgAN	HC	IgAN	HC
	10.1±8.3	14.1±16.9	71.8±21.3	95.5±7.5	3.6±3.4	3.8±6.2	9.0±14.6	5.5±13.8	80.8±11.5	82.3±21.1
